# Organocatalyzed ring-opening copolymerization of α-bromo-γ-butyrolactone with ε-caprolactone for the synthesis of functional aliphatic polyesters – pre-polymers for graft copolymerization

**DOI:** 10.1080/15685551.2018.1550288

**Published:** 2018-11-29

**Authors:** Chen Gao, Chi-Hui Tsou, Chun-Yan Zeng, Li Yuan, Rui Peng, Xue-Mei Zhang

**Affiliations:** a College of Materials Science and Engineering, Sichuan University of Science and Engineering, Zigong, Sichuan Province, China; b Material Corrosion and Protection Key Laboratory of Sichuan Province, Zigong, Sichuan Province, China

**Keywords:** εCL, αBrγBL, DPP, ring-opening polymerization, ATRP, functional aliphatic polyester

## Abstract

Diphenyl phosphate (DPP) was exploited as an organocatalyst to synthesize copolymers by ring-opening polymerization with α-bromo-γ-butyrolactone (αBrγBL) and ε-caprolactone (εCL) as monomers and polyethylene glycol (PEG) as initiator. The conversion rates of monomers and molecular weights of copolymers synthesized under different conditions were determined by ^1^H-NMR. The ^1^H-NMR results showed that the copolymers of αBrγBL and εCL initiated by PEG (PEGCB) were successfully synthesized and the conversions of εCL were relatively high (＞70%), while the conversions of αBrγBL were relatively low (＜26%). The highest molar ratio of αBrγBL to εCL units in these copolymers is 0.17, when the copolymerization was carried out at 100℃ for 17h.

The bromine atoms hanged on the chain of the copolymers PEGCB provide a good opportunity to construct graft copolymers via atom transfer radical polymerization (ATRP). The subsequent grafting of 2-(dimethylamino)ethyl methacrylate (DMAEMA) was conducted by using PEGCB3 as macroinitiator, CuBr/N,N’,N’,N”,N”- pentamethyldiethylenetriamine (PMDETA) as catalysts and toluene/anisole as solvents via ATRP. According to the analysis of ^1^H-NMR, the grafting efficiency, grafting ratio and grafting frequency were 22.4%, 160.7% and 1133.8, respectively.

## 1. Introduction

In recent years, aliphatic polyesters have been widely investigated due to their inherent good degradability, flexibility and biocompatibility. Hence, aliphatic polyesters are widely used to prepare polyurethane [1,2], elastomers [3], drug delivery materials [4,5] and scaffold for tissue engineering [6,7]. Among these, poly(ε-caprolactone) is of great interest due to that εCL is commercially available, biocompatible and readily polymerizable. A commonly used technique to prepare aliphatic polyesters is controlled ring-opening polymerization (CROP) which can construct well-defined polymers. However, up to now, many CROPs of εCL are catalyzed by metal-containing catalysts, such as stannous octoate [Sn(Oct)_2_] [8,9], zinc-containing catalyst [10], iron-containing catalyst [11], magnesium-containing catalyst [12] and aluminum-containing catalyst [13], to name a few. It is attractive that the recent development of metal-free organocatalysts for CROP brings a new horizon of preparing aliphatic polyesters. The organocatalyzed polymerization process could be more suitable to prepare biocompatible materials owing to its low toxicity. Organic Brønsted acids, e.g., sulfuric acid [14], carboxylic acid [15], trifluoromethanesulfonic acid (TfOH) [16,17], methanesulfonic acid (MSA) [18] and phosphoric acids [19] were found to be effective for the CROP of lactones. Although numerous investigations have been carried out, there still exist some shortcomings need to be overcome for the synthesis of aliphatic polyesters using organocatalysts. For instance, strong acidity is necessary to promote the ring-opening polymerization of lactones, however, the strong acidity of organocatalysts may lead to undesirable reactions, such as transesterification.

Recently, diphenyl phosphate (DPP), a weak acid organocatalyst, was found to be effective for the CROP of lactones, such as δ-valerolactone (δVL) and ε-caprolactone (εCL) [20,21]. It is exciting that with the use of DPP, the ring-opening polymerization of lactones could proceed in a living fashion and the molecular weights of polymers were well-controlled. It is worth mentioning that the polymerization catalyzed by DPP can proceed in mild condition (r.t.), which can reduce undesirable side reactions.

Functional aliphatic polyesters provide a diverse range of properties which can be tailored by reactions of functional groups. The routes for the synthesis of functional aliphatic polyesters range from copolymerization with functional monomers and modification to functional initiators. Between these two methods, copolymerization with functional monomers provides a high degree of functionality, together with a high degree of control. Therefore, much effort has been conducted, as early as 1999, εCL with a halogenated motif at the γ-position was synthesized [22]. Subsequently, α-, γ- and ω-substituted εCL were synthesized and used as functional monomers for lipase-catalyzed ring-opening polymerization [23]. However, synthesis of functional monomers often involves multiple steps, which increase the cost and limit the application of aliphatic polyesters. We therefore turned our attention towards a straightforward and commercially available functional monomer, α-bromo-γ-butyrolactone (αBrγBL).

As early as 1932, ring-opening polymerization of γ-butyrolactone (γBL) has been investigated, when Carothers *et al*. discovered that unsubstituted γBL was unable to polymerize [24]. Subsequently, some theoretical calculations reconfirmed this result [25,26]. The same behavior was also observed for αBrγBL. High molecular weight homopolymer is unable to be obtained by ring-opening polymerization of αBrγBL. Not considering polymers synthesized under extremely high pressure [27] or low polymerization temperatures [28]. This is due to αBrγBL’s thermodynamic propensity to ring-close. However, this can be circumvented by copolymerization of αBrγBL with monomers having high ceiling temperature (T_c_), i.e., thermodynamic favoring polymerization, such as εCL. Hence, copolymers of γBL and εCL with high number average molecular weight can be obtained at relative high temperature [29]. It is worth mentioning that αBrγBL’s inability to form a homopolymer should result in isolated units along the polymer chain during copolymerization. This provides a good opportunity to initiate graft polymerization by controlled radical polymerization, e.g., ATRP. The isolated initiating sites are favourable to reduce the grafting steric hindrance and increase the monomer conversion in graft polymerization.

Poly[(2-dimethylamino)ethyl methacrylate] (PDMAEMA) is a kind of pH- and temperature-responsive polymer [30,31] that can be synthesized by ATRP initiated by chlorine or bromine functional group. PDMAEMA is hydrophilic when p*K*a is lower than 7 due to its amine groups are protonated. In this condition, as a kind of cationic polymer, PDMAEMA can be used for combining and delivering genes [32]. At the same time, PDMAEMA is hydrophilic when temperature is lower than its lower critical solution temperature (LCST) and hydrophobic when temperature is higher than its LCST. As a hydrophobic polymer, PCL is one of the most promising biocompatible and biodegradable polymers with high drug permeability. As a hydrophilic polymer, PEG exhibits many unique characteristics, such as biocompatibility and flexibility, which enable it to be valuable in biomedical field, such as drug delivery systems and tissue engineering. Both PEG and PCL have been approved by the United States Food and Drug Administration (FDA) and widely used as biomedical materials [33].

Our aim is to produce amphiphilic functional copolymers based on αBrγBL and εCL by ring-opening polymerization. The hypothesis is that DPP will provide a broard range of polymerization temperatures due to its high catalytic activity. It is anticipated that different conversions of αBrγBL and εCL should be achieved at different temperatures and during different polymerization time. In this study, these factors of ring-opening copolymerization of αBrγBL with εCL will be investigated. Subsequently, ATRP of DMAEMA by using this bromine-containing multifunctional aliphatic polyester as macroinitiator was attempted. If successful this would provide the possibility to produce a diverse range of graft copolymers with degradable backbones by convenient synthetic route. This abundance is ascribed to a great diversity of vinyl monomers with various functionality which are liable to ATRP [34–37].

CROP and ATRP are both effective methods for constructing polymers. However, how to combine the two methods to synthesize well-defined polymers conveniently is an important issue. αBrγBL is a kind of cyclic monomer containing bromine functional group which can provide a platform to combine CROP and ATRP. Although αBrγBL holds low activity in CROP due to its low *T_c_*, CROP of αBrγBL catalyzed at mild temperature is still attractive. Recent advances in CROP catalyzed by organocatalysts have revealed high reactivity at ambient temperature. Diphenyl phosphate (DPP), a representative organocatalyst, can catalyze the CROP of lactones in mild condition to produce polyesters with well-defined structure. The copolymerization of εCL with αBrγBL catalyzed by DPP was mentioned by Albertsson *et al*., with the molar ratio of αBrγBL to εCL repeating units in copolymer is 0.05 when the polymerization was conducted at 30 ℃ for 24 h [38]. However, as far as we are concerned, the influence of polymerization temperature and time on the copolymerization of αBrγBL with εCL has not been investigated till now. In this study, it reveals that the CROP reactivities of αBrγBL and εCL are affacted by temperature and time in different extent.

## 2. Experimental

### 2.1. Materials

ε-Caprolactone (99%; Aladdin) was dehydrated by calcium hydride and filtrated by using microfilter (0.45 μm) before use. Poly(ethylene glycol) (M_n_ = 2 K; Aladdin) was dried by azeotropic distillation in the presence of toluene. Diphenyl phosphate (97%; Energy Chemical), cuprous bromide (99%; Aladdin), N,N’,N’,N”,N”- pentamethyldiethylenetriamine (PMDETA) (99%; Aladdin), 2-(dimethylamino)ethyl methacrylate (DMAEMA) (99%; Aladdin), α-bromo-γ-butyrolactone (αBrγBL) (98%; J&K Chemicals) were used without further purifications, toluene (AR; Chongqin Chuandong Chemical Group Co. Ltd.), ether (AR; Chongqing Chuandong Chemical Group Co. Ltd.), methylene dichloride (AR; Chengdu Jingshan Chemical Reagent Co. Ltd.), n-hexane (AR; Chengdu Kelong Chemical Reagent Factory) and basic alumina (100–200 mesh; Aladdin) were used as received.

### 2.2. Measurements

^1^H-NMR spectra were recorded on a Bruker Avance DMX 500 spectrometer using CDCl_3_ as solvent and tetramethylsilane as internal reference. The molecular weight and molecular weight distribution were determined by size exclusion chromatographic (SEC). The SEC system consisted of a Waters degasser, a Waters 1525 HPLC pump with 717 plus autosampler, Waters 2410 RI detector and columns: Styragel, HT 3; HT 4. The calibration was performed with commercial polystyrene standards. Tetrahydrofuran (THF) was used as the mobile phase at a flow rate of 1.0 mL/min at 40℃.

### 2.3. Sythesis of PEGCB by CROP

A typical procedure for the polymerization is as follows: PEG2k (500 mg, 0.25 mmol) and 10 ml toluene were mixed in a 100 ml Schlenk flask equipped with a magnetic stirring bar. Then the flask was put into an oil bath thermostated at 140 ℃ to dehydrate the PEG2k with toluene azeotropic method. Then α-bromo-γ-butyrolactone (2.2 ml, 23.9 mmol), ε-caprolactone (3.8 ml, 34.3 mmol) and diphenyl phosphate(378 mg, 1.5 mmol) were added into the flask under argon atmosphere. Then the flask was thermostated at 60 ℃ for 7.5 h. The crude product was dissolved in CH_2_Cl_2_ and poured into diethyl ether to precipitate the final product, which was dried in vacuum to constant weight. Yield: 3.50 g (41.9 %). ^1^H-NMR (PEGCB3, CDCl_3_): δ = 4.34 ppm (-C*H*BrCH_2_CH_2_O-), 4.20 ppm (-CHBrCH_2_C*H*_2_O-), 4.06 ppm (-COCH_2_CH_2_CH_2_CH_2_C*H*_2_O-), 3.65 ppm (-OC*H*_2_C*H*_2_O-), 2.48 ppm (-CHBrC*H*_2_CH_2_O-), 2.31 ppm (-COC*H*_2_CH_2_CH_2_CH_2_CH_2_O-), 1.65 ppm (-COCH_2_C*H*_2_CH_2_C*H*_2_CH_2_O-), 1.39 ppm (-COCH_2_CH_2_C*H*_2_CH_2_CH_2_O-).

### 2.4. Sythesis of PEGCB-g-PDMAEMA by ATRP

A typical procedure for the synthesis of PEGCB-g-PDMAEMA is as follows: a Schlenk flask placed a stirring bar was deoxygenated by three pump-fill cycles. Then CuBr (0.7 mmol，100.8 mg), PMDETA (1.4 mmol，0.292 mL) and toluene/anisole (v:v = 1:1, 4 ml) were added into the flask under argon atomosphere. After stiring for 15 min, DMAEMA（4.72 ml, 28 mmol） and PEGCB3（0.5 g, 0.44 mmol Br）were added into the flask to be homogeneously mixed. The mixed solution was bubbled with argon for about 15 min to deoxygenate the solution. Then the flask was put into an oil bath thermostated at 70 ℃ for 30 h. The solution was diluted by adding 30 ml THF and passed through a basic alumina column to remove the copper salt. The graft copolymer was recovered by condensing the solution and precipitating with n-hexane and dried in vacuum. Yield: 1.82 g (37.1 %). ^1^H-NMR (PEGCB3-g-PDMAEMA, CDCl_3_): δ = 4.08 ppm (-COCH_2_CH_2_CH_2_CH_2_C*H*_2_O- and – OC*H*_2_CH_2_N(CH_3_)_2_), δ = 3.83 ppm (-COCHRCH_2_C*H*_2_O-), δ = 3.66 ppm (-OC*H*_2_C*H*_2_O-), δ = 2.59 ppm [–OCH_2_C*H*_2_N(CH_3_)_2_], δ = 2.30 ppm [–OCH_2_CH_2_N(C*H*_3_)_2_, -COC*H*_2_CH_2_CH_2_CH_2_CH_2_O- and -COC*H*RCH_2_CH_2_O-], δ = 1.93 ppm [-CHC*H*_2_CR’(CH_3_)COO-, atactic], δ = 1.83 ppm [-CHC*H*_2_CR’(CH_3_)COO-, syndiotactic and -COCHRC*H*_2_CH_2_O-], δ = 1.66 ppm (-COCH_2_C*H*_2_CH_2_C*H*_2_CH_2_O-), δ = 1.40 ppm (-COCH_2_CH_2_C*H*_2_CH_2_CH_2_O-), δ = 1.07 ppm [-CHCH_2_CR’(C*H*_3_)COO-, atactic], δ = 0.92 ppm [-CHCH_2_CR’(C*H*_3_)COO-, syndiotactic].

## 3. Results and discussion

### 3.1. Elucidating the copolymerization behavior of αBrγBL with εCL

In the 1930s, Carothers stated that ‘the γ-lactones and other five-membered cyclic esters show no tendency to polymerize, and no corresponding polymers are known’. Later, this statement was proved by experiments. Hence, αBrγBL, as a five-membered lactone, is also hard to homopolymerize. Albertsson has reported that no homopolymerization of αBrγBL was observed after 20 h with Sn(Oct)_2_ as catalyst at 110 ℃ [38]. However, polymerization of αBrγBL can be circumvented by copolymerization with other cyclic monomer, i.e., εCL.

**Figure 1.**
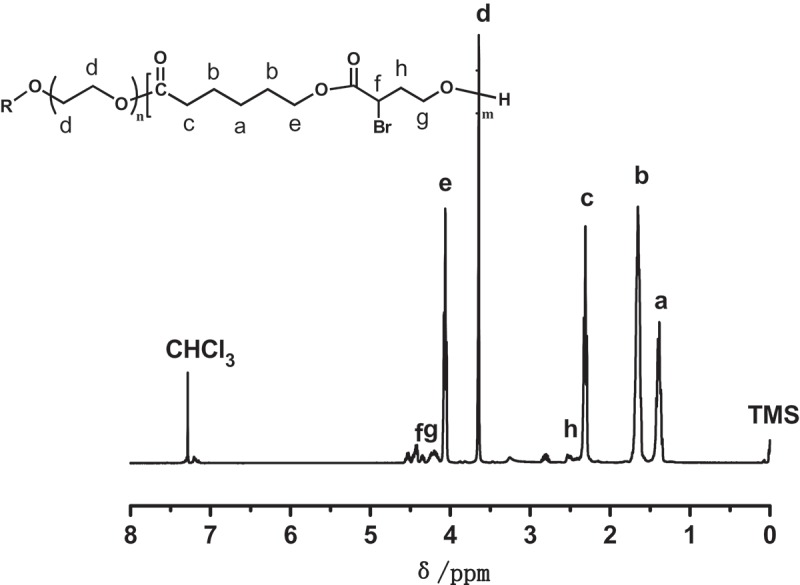
^1^H-NMR spectra of the copolymer PEGCB3.

**Scheme 1.**
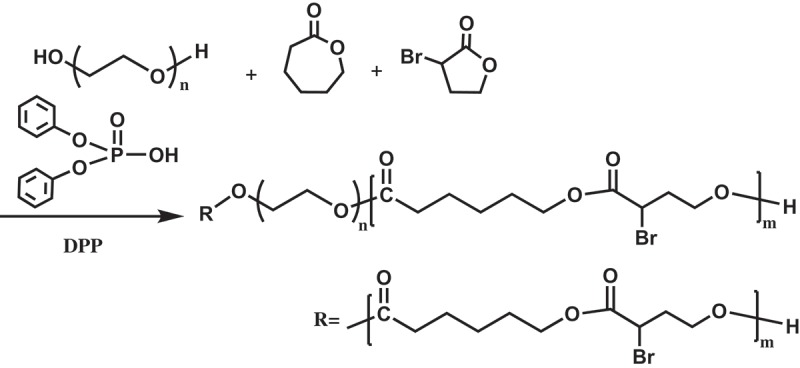
Synthesis of PEGCB copolymers by ring-opening copolymerization of εCL with αBrγBL using PEG as initiator and DPP as organocatalyst.

In this study, copolymerizations of αBrγBL with εCL at different temperatures and for different time were conducted. The copolymerization process is depicted in Scheme 1. The ^1^H-NMR spectrum of a representative copolymer PEGCB3 is shown in Figure 1.

It clearly shows that besides the initiator proton signals of PEG chains (*H* ^d^), there are aliphatic polyester proton signals of PεCL(*H* ^a,b,c,e^) and PαBrγBL (*H* ^f,g,h^). This suggests that the copolymerization of αBrγBL with εCL was proceeded successfully. The integral ratio of peak areas of *H* ^d^ and *H* ^e^ (*I* ^d^/*I* ^e^) is 0.78, which is not far from the theoretical value (*I* ^d^/*I* ^e^ = 0.66). It indicates the high conversion of εCL (84.8%). The integral ratio of peak areas of *H* ^d^ and *H* ^g^ (*I* ^d^/*I* ^g^) is 5.72, which is much higher than the theoretical value (*I* ^d^/*I* ^g^ = 0.94). This reveals the conversion of αBrγBL is relatively low (16.5%), thus confirming that αBrγBL holds low activity, which has been reported in the literatures [24]. By calculating from the ^1^H-NMR spectrum of PEGCB3, the molecular weight of PEGCB3 was about 17,800 and there were about 15.8 αBrγBL repeating units on every copolymer chain. The compositions of PEGCB1-PEGCB8 copolymers determined by ^1^H-NMR are shown in Table 1. The ^1^H-NMR spectrums of polymers PEGCB1-PEGCB8 except PEGCB3 are shown in Supporting Information Figures S1–S7.

**Table 1.** Compositions of the PEGCB copolymers under different polymerization conditions.

Polymer	Temp. (℃)	Time (h)	*M*_t_^a^	*M*_NMR_^b^	Conv. (εCL)	Conv. (αBrγBL)	*n_BL_/n_CL_*^c^	*n_BL_/n_CL_*^d^
PEGCB1	25	17	33,400	18,300	93.9%	10.1%	0.69	0.07
PEGCB2	25	29	33,400	20,900	99.0%	21.4%	0.69	0.15
PEGCB3	60	7.5	33,400	17,800	84.8%	16.5%	0.69	0.14
PEGCB4	60	17	33,400	21,500	＞99.0%	16.1%	0.69	0.10
PEGCB5	60	29	33,400	18,000	89.4%	13.0%	0.69	0.10
PEGCB6	100	6	33,400	16,200	84.8%	10.4%	0.69	0.09
PEGCB7	100	17	33,400	22,700	＞99.0%	25.7%	0.69	0.17
PEGCB8	100	29	33,400	16,000	73.8%	15.3%	0.69	0.14

^a^ Theoretical number average molecular weight.

^b^ Number average molecular weight determined by ^1^H-NMR.

^c^ Feed ratio of αBrγBL and εCL

^d^ The ratio of αBrγBL and εCL repeating units in copolymers determined by ^1^H-NMR.

Table 1 summarizes the polymerization results. ^1^H-NMR analysis revealed that the conversions of εCL were relatively high (＞70%) and the conversions of αBrγBL were relatively low (＜26%). The low conversions of αBrγBL would be considered a drawback if it acted as a property-alternating monomer, but its main purpose is to act as an initiator for ATRP, the incorporated amount of αBrγBL is enough. The low conversion of αBrγBL provides a more possibility to form isolated units, thus decreasing the steric hindrance of sequential ATRP graft copolymerization.

**Figure 2.**
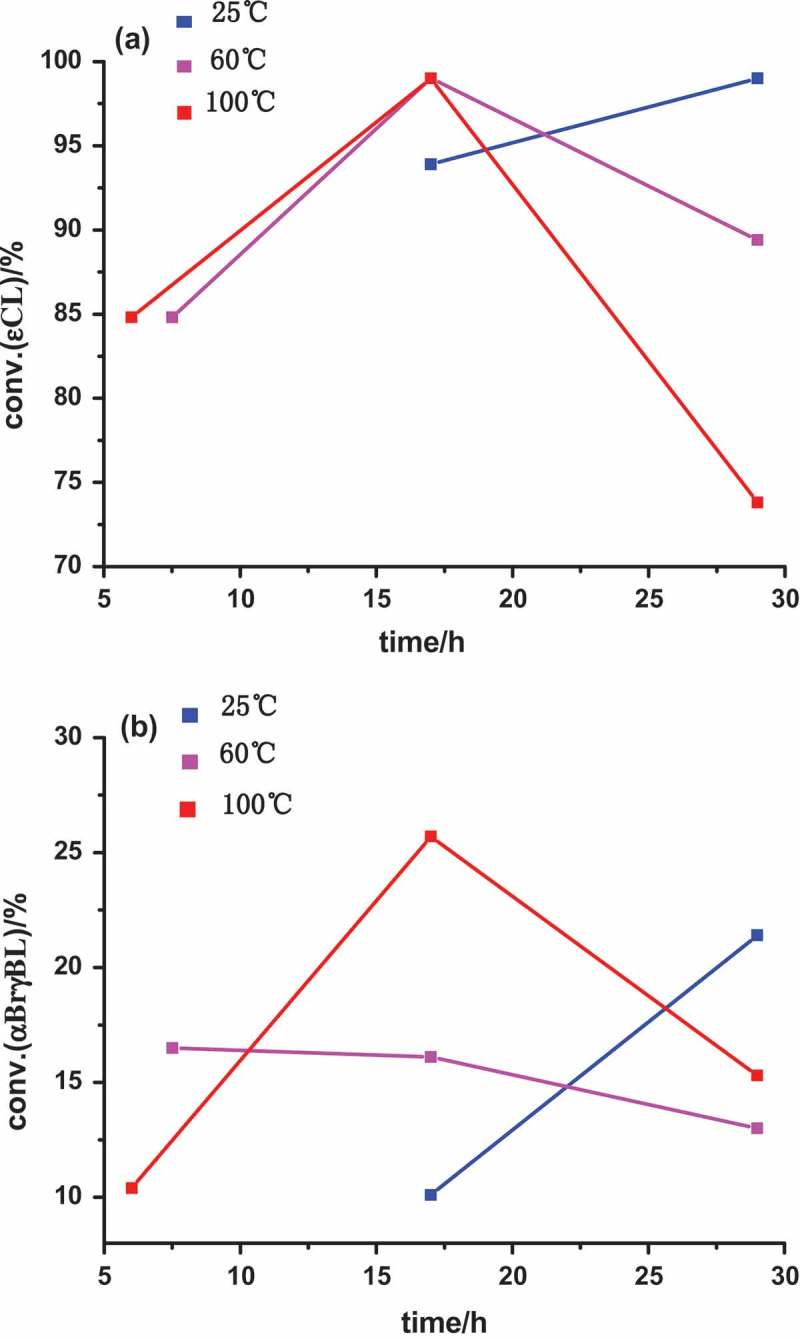
(a) relationship of εCL monomer conversion [conv.(εCL)] and time. (b) relationship of αBrγBL monomer conversion [conv.(αBrγBL)]and time.

The relationship of monomer conversions and polymerization time are shown in Figure 2. It shows that the monomer conversions of both εCL and αBrγBL are increased with the increasing of polymerization time (from 17 h to 29 h) at 25℃. However, the monomer conversions of both εCL and αBrγBL are decreased with the increasing of polymerization time (from 17 h to 29 h) at higher polymerization temperatures (60℃ or 100℃). This might be attributed to the severer degradation of polyesters at higher temperatures. The molar ratio of αBrγBL and εCL repeating units in copolymers could attain 0.17 when polymerization was conducted at 100℃ for 17 h (Table 1, entry7) and there were about 24.5 αBrγBL repeating units on every copolymer chain of PEGCB7. This provides adequate active sites for the sequential grafting polymerization.

### 3.2. Elucidating the graft copolymerization of DMAEMA by ATRP

**Figure 3.**
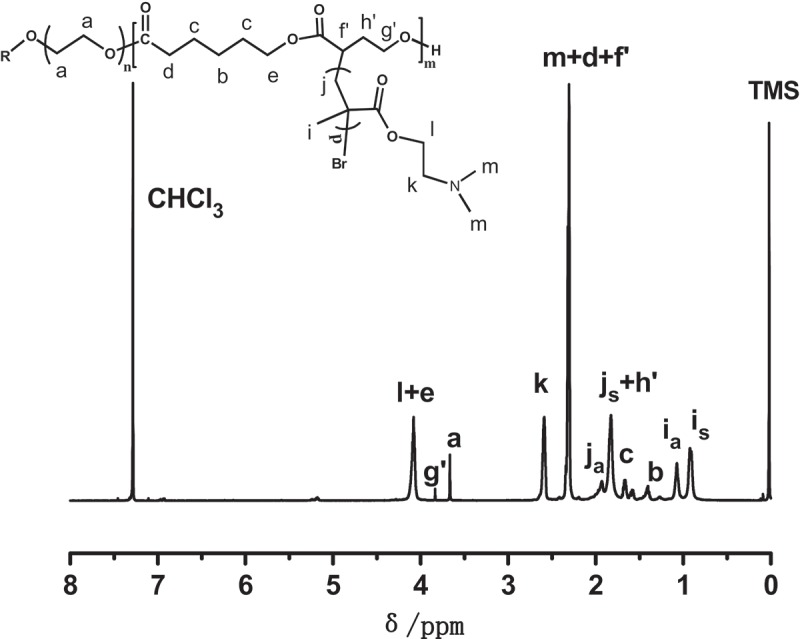
^1^H-NMR spectra of the graft copolymer PEGCB3-g-PDMAEMA.

**Scheme 2.**
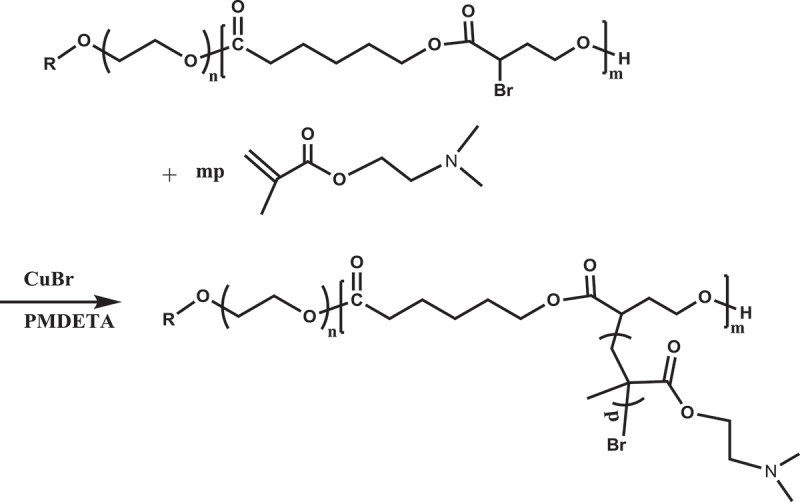
Synthesis of PEGCB-g-PDMAEMA graft copolymers by ATRP.

The graft copolymerization of DMAEMA is depicted in Scheme 2. Successful formation of graft copolymer PEGCB3-g-PDMAEMA was verified by ^1^H-NMR (Figure 3). Resonances originating from both PEGCB3 and PDMAEMA segments were observed.

From the ^1^H-NMR spectra shown in Figures 1 and 3, it is obvious that the signal of methylene protons of αBrγBL units in copolymer [Figure 1 (*H* ^h^)] disappeared completely. Similarly, the signal of methylene protons of αBrγBL units in copolymers [Figure 1 (*H* ^g^)] also disappeared completely and a corresponding signal [Figure 3 (*H* ^g’^)] appeared at 3.83 ppm. This is because the electron-withdrawing bromine atoms have migrated to the end of the PDMAEMA graft chains, thus rendering the signals of methylene protons move to the higher magnetic field. The notations a and s, added to j and i, designate whether the monomer sequence in the PDMAEMA graft chain is atactic or syndiotactic respectively [39]. There is no proton signals at 5.70ppm and 6.10ppm due to the methylene protons of DMAEMA monomer. This suggested that there is no residual DMAEMA monomer in the graft copolymer. The number of DMAEMA repeating units is about 813 by comparing the integrals of peaks k and a, which was ascribed to PDMAEMA and PEG, respectively. However, according to the ^1^H-NMR of PEGCB3, the integral ratio of peak areas of α-proton originate from αBrγBL monomer and α-proton originate from αBrγBL repeating units in PEGCB3 copolymer is 3.48. The bromine atoms of the αBrγBL monomer (δ_α-proton_ = 4.55 ppm, Figure 1) may also initiate the ATRP of DMAEMA. Hence, there were only about 11.5 DMAEMA repeating units on every graft chain determined by ^1^H-NMR. The number average molecular weight of graft copolymer PEGCB3-g-PDMAEMA is about 46,400 (Table 2).

**Table 2.** Results of graft copolymerization of DMAEMA by ATRP.

Macroinitiator	*n*_Br_^a^	*N*_Br_^b^ (mmol/g)	*R*_t_ ^c^	*R*_NMR_	*M*_n,NMR_^e^	*M*_n,SEC_ ^f^
PEGCB3	15.7	0.88	63.6	11.6	46,400	24,400

^a^ Number of bromine atoms in every PEGCB3 chain determined by ^1^H-NMR. Calculated from *n*_Br_ = (*m*_αBrγBL_ */m*_PEG_)*(*M*_PEG_ */M*_αBrγBL_)*Conv. _αBrγBL_.

^b^ Content of bromine atoms in PEGCB3 determined by ^1^H-NMR. Calculated from *N*_Br_ = (1/*M*_PEGCB3_)*n_Br_.

^c^ Theoretical molar ratio of DMAEMA monomer to bromine atoms. Calculated from *R_t_ *= *n*_DMAEMA_/(*m*_PEGCB3_*N_Br_).

^d^ Molar ratio of DMAEMA units to bromine atoms in graft copolymer determined by ^1^H- NMR. Calculated from *R*_NMR_ = *U*_DMAEMA_*(*n*_g_/(*n*_g_+*n*_h_))/*n*_Br_. *U*_DMAEMA_ represents the number of DMAEMA units in both graft chains and homopolymer chains. *n*_g_ and *n*_h_ represent the number of initiation points in graft polymerization and homopolymerization, respectively.

^e^ Number average molecular weight of graft copolymer determined by ^1^H-NMR. Calculated from *M*_n,NMR_ = *M*_PEGCB3_+* n*_Br_* *R*_NMR_*M_DMAEMA_.

^f^ Number average molecular weight determined by SEC

**Figure 4.**
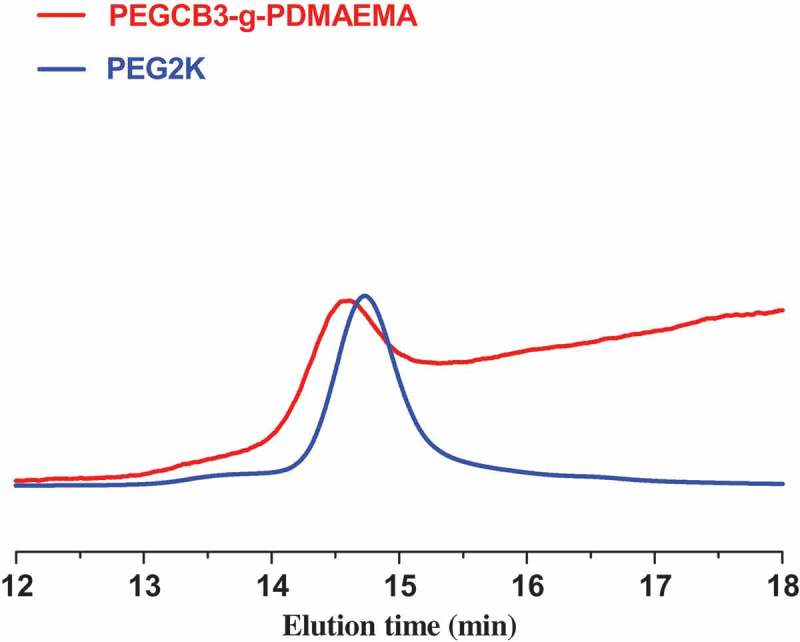
SEC traces of graft copolymer PEGCBD3-g-PDMAEMA and PEG2K.

As shown in Figure 4, the SEC chromatogram of PEGCB3-g-PDMAEMA shows unimodal peak and narrow molecular weight distribution (d = 1.08), confirming the controlled nature of ATRP grafting. However, according to the results of ^1^H-NMR, the final graft polymerization product contained a mixture of graft copolymer and the homopolymer of DMAEMA. This contradiction is arised from PDMAEMA homopolymers are known to be difficult to characterize by SEC due to the adsorption of the amine group onto the column [40]. Although the graft copolymers of DMAEMA also have amine group, homopolymers are more likely to be adsorbed by the column due to their higher amine group density. This can account for the unimodal character of the trace in SEC of PEGCB3-g-PDMAEMA.

After CROP and ATRP, the SEC trace is shifted towards shorter elution time region, representing higher molecular weight, indicating the actual polymerization process. The number average molecular weight of PEGCBD3-g-PDMAEMA determined by SEC is smaller than that determined by ^1^H-NMR, as shown in Table 2. This is due to the fact that the hydrodynamic volume of graft polymer is smaller than that of linear polymer with the same molecular weight. Besides, the use of polystyrene as calibrating standards can also account for the large difference in molar masses.

Three different grafting parameters were calculated according to a method reported by Huang and Sundberg [41]. The grafting efficiency (GE) is defined as the weight percent of polymer that is grafted. The GE is calculated by the mass of the PDMAEMA that is grafted divided by the total mass of PDMAEMA produced, both grafted and homopolymer (Equation 1)

(1)
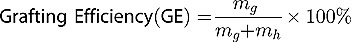

In Equation 1, *m_g_* represents the mass PDMAEMA in graft copolymer, *m_h_* represents the mass of PDMAEMA homopolymer.

The grafting ratio (GR) is the average amount of grafts per unit mass of backbone polymer. Equation 2 shows that the GR equals the amount of grafted PDMAEMA divided by the mass of the PEGCB backbone.

(2)
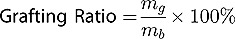

In Equation 2, *m_g_* represents the mass PDMAEMA in graft copolymer, *m_b_* represents the mass of PEGCB backbone.

The grafting frequency (GF) is defined as the number average molecular weight of backbone between graft points. Equation 3 shows that the GF equals the number average molecular weight of the backbone multiply the number of PEGCB backbone chains and divided by the number of grafted PDMAEMA chains.

(3)
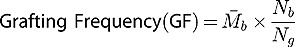

In Equation 3, 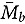 represents the number average molecular weight of the PEGCB backbone. *N_b_* and *N_g_* represents the number of PEGCB backbone chains and the number of grafted PDMAEMA chains, respectively.

All of the grafting parameters were calculated and summarized in Table 3.

**Table 3.** Grafting parameters of graft copolymerization of DMAEMA by ATRP.

Macroinitiator	Grafting Efficiency (%)	Grafting Ratio (%)	Grafting Frequency
PEGCB3	22.4	160.7	1133.8

## 4. Conclusions

Organocatalyzed ring-opening copolymerizations of α-bromo-γ-butyrolactone (αBrγBL) with ε-caprolactone (εCL) were conducted by using DPP as catalyst and PEG as macroinitiator. To visualize how the conversions of αBrγBL and εCL are affected by polymerization temperature and time, several reactions under different conditions were conducted. The results showed that the conversions of εCL were relatively high (＞70%) and the conversions of αBrγBL were relatively low (＜26%). When ring-opening polymerizations were conducted at ambient temperature, higher conversions of both εCL and αBrγBL were attained at longer time(29h). However, when polymerizations were conducted at higher temperatures(60℃ or 100℃), the highest conversions of both εCL and αBrγBL were attained at a proper time (17 h). The The number of αBrγBL repeating units on copolymer chain could reach 24.5 and the molar ratio of αBrγBL and εCL repeating units could reach 0.17 when copolymerization was conducted at 100℃ for 17 h. The low tendency to form homopolymer of αBrγBL provides the opportunity to form isolated units of αBrγBL on copolymer chains, which could reduce the steric hindrance of grafting copolymerization and improve the initiating efficiency of bromine groups. The subsequent grafting of DMAEMA via ATRP with PEGCB3 as macroinitiator was conducted. According to the analysis of ^1^H-NMR, the number average molecular weight of PEGCB3-g-PDMAEMA graft copolymer is about 46,400, together with high initiation efficiency of the αBrγBL repeating units. The grafting efficiency, grafting ratio and grafting frequency were 22.4%, 160.7% and 1133.8, respectively.
