# Supplementary material for: Organocatalyzed ring-opening copolymerization of α-bromo-γ-butyrolactone with ε-caprolactone for the synthesis of functional aliphatic polyesters – pre-polymers for graft copolymerization
Source: Des Monomers Polym. 2018 Nov 29;21(1):193–201. doi: 10.1080/15685551.2018.1550288 (PMC6282472; doi:10.1080/15685551.2018.1550288)
Supplement: Supplemental Material [file TDMP_A_1550288_SM4329.doc]

**Supporting Information for**

**Organocatalyzed ring-opening copolymerization of α-bromo-γ-**

**butyrolactone with ε-caprolactone for the synthesis of functional aliphatic polyesters – pre-polymers for graft copolymerization**

Chen Gao^a,b^, Chi-Hui Tsou^a,b^, Chun-Yan Zeng^a,b^, Li Yuan^a,b^, Rui Peng^a^, Xue-Mei Zhang^a,b^,***

*^a^College of Materials Science and Engineering, Sichuan University of Science and Engineering, Zigong, Sichuan Province, China*

*^b^Material Corrosion and Protection Key Laboratory of Sichuan Province, Zigong, Sichuan Province, China*

* Corresponding author

E-mail address: 798538268@zju.edu.cn (Xue-Mei Zhang)

Fig. S1 ^1^H NMR spectrum of PEGCB1.

Fig. S2 ^1^H NMR spectrum of PEGCB2.

Fig. S3 ^1^H NMR spectrum of PEGCB4.

Fig. S4 ^1^H NMR spectrum of PEGCB5.

Fig. S5 ^1^H NMR spectrum of PEGCB6.

Fig. S6 ^1^H NMR spectrum of PEGCB7.

Fig. S7 ^1^H NMR spectrum of PEGCB8.
